# Exploring the antiviral potential of shikimic acid against Chikungunya virus through network pharmacology, molecular docking, and *in vitro* experiments

**DOI:** 10.3389/fvets.2025.1524812

**Published:** 2025-01-23

**Authors:** Jialiang Xin, Xingxing Song, Haohong Zheng, Wenjing Li, Yuyang Qin, Wei Wang, He Zhang, Guangneng Peng

**Affiliations:** ^1^Key Laboratory of Animal Disease and Human Health of Sichuan Province, College of Veterinary Medicine, Sichuan Agricultural University, Chengdu, China; ^2^College of Animal Science and Technology, Guangxi University, Nanning, China; ^3^Institute of Virology, Wenzhou University, Wenzhou, China; ^4^Changchun Veterinary Research Institute, Chinese Academy of Agricultural Sciences, Changchun, Jilin, China

**Keywords:** CHIKV, shikimic acid, antiviral, network pharmacology, molecular docking

## Abstract

Chikungunya virus (CHIKV) is an arbovirus that can lead to chronic arthritis and significantly diminish the quality of life of patients. Given the expanding global prevalence of CHIKV and the absence of specific antiviral therapies, there is an urgent need to explore effective treatment options. This study aimed to evaluate the antiviral effects of shikimic acid (SA) against CHIKV through a combination of network pharmacology, molecular docking, and *in vitro* assays. Network pharmacology analysis identified 26 potential targets through which SA could inhibit CHIKV, including key pathogenic targets such as TNF, IL-6, and MAPK3. This hypothesis was further supported by molecular docking. The molecular docking analysis revealed that SA could interact with multiple CHIKV-related targets, including EGF, with vina scores generally lower than −6, indicating a high propensity for stable complex formation. The results also suggested that SA could potentially disrupt the IL-17 signaling pathway by engaging with various targets to form complexes. *In vitro* experiments confirmed that SA significantly enhanced the viability of 293T and BHK-21 cells infected with CHIKV by ~25% and reduced viral load by over 20% at concentrations ranging from 1,000 to 31.25 μM. Additionally, SA was found to markedly downregulate the expression of CHIKV-related attachment factors ACTG1, TSPAN9, and TIM-1 in 293T cells infected with CHIKV. Furthermore, RT-qPCR analysis demonstrated that SA effectively decreased the expression of NFKB1, PTGS2, RELA, and EGF related to the IL-17 signaling pathway. In conclusion, these findings indicate that SA is a promising candidate for developing treatment strategies targeting CHIKV with good clinical application value.

## 1 Introduction

Chikungunya Fever (CHIKF) is a zoonotic disease caused by the Chikungunya virus (CHIKV). It is characterized by symptoms such as fever, headache, nausea, muscle pain, joint pain, and polyarthritis during its acute phase (1). Following the onset of initial symptoms, many patients may develop a chronic joint condition characterized by severe pain and deformity, lasting from months or even years (2). This chronic state significantly diminishes quality of life for patients, imposes substantial demands on healthcare systems and resulting in economic consequences for individuals and communities (3). CHIKV is primarily transmitted by mosquitoes and is prevalent t in over half of the world's regions, with an estimated 4 million cases reported annually (4, 5). Despite its considerable public health implications, treatment options remain limited. Current therapies, such as nonsteroidal anti-inflammatory drugs (NSAIDs) and disease-modifying antirheumatic drugs (DMARDs), are designed to alleviate joint pain associated with CHIKV; however, they come with potential side effects. Prolonged use of NSAIDs can lead to gastric ulcers, particularly in individuals with pre-existing blood disorders (6). Additionally, DMARDs like chloroquine can induce neurological symptoms without significantly reducing viremia or viral load (7). There is an critical need for safe and effective strategies to manage CHIKV symptoms and address this significant public health challenge.

Shikimic acid (SA), derived from the Chinese herb star anise, is a promising therapeutic agent for alleviating symptoms in patients infected with CHIKV. Although the precise anti-CHIKV activity of SA has yet to be definitively established, its broad range of biological activities makes it a candidate of interest. Research indicates that SA may protect cartilage by restoring disrupted autophagy and inhibiting the MAPK/NF-κB signaling pathway (8). Additionally, SA has demonstrated analgesic effects, significantly reducing inflammatory pain triggered by TNF-α (9). Given SA's favorable safety profile, it could serve as a viable long-term treatment for symptoms associated with CHIKV infection.

Network pharmacology, which integrates systems biology and network informatics, provides a comprehensive approach to developing antiviral strategies (10). It posits that complex diseases arise from disruptions in biological networks rather than from single-gene mutations. This holistic method facilitates the analysis of compound effects throughout the body, aiding in the identification of therapeutic targets, enhancing drug efficacy, and minimizing adverse effects (11). Molecular docking, a computational technique that models molecular-protein interactions at the atomic level, accurately predicts binding affinities and serves as a valuable, cost-effective tool in drug design and the elucidation of biochemical pathways (12). This study aimed to identify potential molecular targets and signaling pathways related to the treatment of CHIKV using SA. Molecular docking and *in vitro* experiments were conducted to validate the effects of SA on CHIKV treatment.

## 2 Materials and methods

### 2.1 Cell lines, viruses and compounds

293T and BHK-21 cells, obtained from the China Center for Type Culture Collection (CCTCC), were cultured in Dulbecco's Modified Eagle Medium (DMEM). The medium was supplemented with 10% heat-inactivated fetal bovine serum (FBS), 100 units/ml penicillin, and 100 mg/ml streptomycin.

The Chikungunya virus (GenBank: MT933041.1) was kindly provided by the Changchun Veterinary Research Institute and was stored at −80°C until required for use. Shikimic acid, with a purity ≥99.15%, was purchased from MedChemExpress (CAS No.: 138-59-0). It was diluted in distilled H_2_O to a final concentration of 100 mM.

### 2.2 Network pharmacology-based analysis

#### 2.2.1 Targets acquisition of disease and compound

The keywords “Chikungunya virus” and “Chikungunya fever” were separately used to search seven databases: DisGeNET (https://www.disgenet.org/), GeneCards (https://www.genecards.org/), OMIM (https://omim.org/), MalaCards (https://www.malacards.org), TTD (http://db.idrblab.net/ttd/), STRING (http://cn.string-db.org), and CTD (http://ctdbase.org) to identify disease-related targets, focusing specifically on *Homo sapiens*. Identified disease-related targets were then consolidated by removing duplicates.

The targets related to SA were searched by utilizing the keywords “Shikimic acid” and “138-59-0” across multiple databases: TCMSPS (https://old.tcmsp-e.com/tcmsp.php), PharmMapper Server (http://www.lilab-ecust.cn/pharmmapper/), CTD, Swiss Target Prediction (http://www.swisstargetprediction.ch/), BindingDB (http://www.bindingdb.org/bind/index.jsp), STRING, and TTD, with a focus on *Homo sapiens*. The targets identified from these sources were combined, and any duplicate entries were removed.

#### 2.2.2 Disease and disease-compound intersection target analysis

Venn analysis was conducted to identify common targets between SA and disease-related targets. These targets, along with the common ones, were then imported into STRING with *Homo sapiens* specified and a minimum score confidence of ≥0.9 to obtain protein-protein interaction (PPI) data. Cytoscape 3.9.1 was used to create the PPI network diagram, adjusting node size and color based on degree. The CytoNCA plugin in Cytoscape identified core targets among the disease-related targets. MCODE was applied for cluster analysis of CHIKV targets. DAVID was used for KEGG analysis, and the top 10 pathways with the lowest *p*-values were selected for visualization (http://www.bioinformatics.com.cn/).

#### 2.2.3 Molecular docking validation

The 3D structures of the target proteins were retrieved exclusively from the PDB (https://www.rcsb.org), focusing on “Homo sapiens.” We selected high-resolution (< 3 A) X-ray crystallography structures associated with the top 10 significant targets.

The 2D structure of SA was obtained from PubChem (https://pubchem.ncbi.nlm.nih.gov/). These protein and ligand structures were then imported into CB-DOCK2 (http://cadd.labshare.cn/cb-dock2/php/index.php) for Structure-based Blind Docking analysis. The docking results were visualized, prioritizing complexes with the lowest Vina scores.

### 2.3 Experimental validation

#### 2.3.1 Determination of concentration of cytotoxicity 50%

To determine the concentration of cytotoxicity 50% (CC50) of SA on BHK-21 and 293T cells. Cells were plated in 96-well plates. Subsequently, the BHK-21 or 293T cells were exposed to different concentrations of SA prepared in DMEM supplemented with 2% FBS. After a 48-h incubation period, cell viability was assessed using the Cell Counting Kit-8 (CCK-8) assay (Vazyme, A311-01, China).

#### 2.3.2 Dose-dependent anti-chikungunya assay

The antiviral effects of SA against CHIKV were evaluated using different doses. Briefly, 293T or BHK-21 cells were seeded in 96-well plates and subsequently infected with CHIKV at an MOI of 0.01 for 2 h in presence of different concentrations of SA. After a 24-h incubation, cell viability was assessed using the CCK-8 assay. The formula for calculating cell viability is as follows: Viability (%) = [(AsOD450 nm – AbOD450 nm)/(AcOD450 nm – AbOD450 nm)] × 100% (As is the absorbance value of the experimental group, Ab is the absorbance value of the blank group, Ac is the absorbance value of the control group).

To further evaluate the protective effect of SA on CHIKV-infected cells, 293T cells were cultured in 12-well plates and infected with CHIKV at an MOI of 0.01 for 2 h. Subsequently, the optimal concentration of SA was then added. RNA extraction and quantification of TNF-α, IL-6, and IL-1β by RT-qPCR were performed 24 h post-infection. Relative RNA levels were determined using the 2^−ΔΔ*Ct*^ method, with primer sequences listed in Supplementary Table 1.

#### 2.3.3 Antiviral replication experiments

To evaluate the protective effect of SA on 293T and BHK-21 cells against CHIKV replication, cells were pre-treated with SA (1000 μM, 500 μM, 250 μM) for 2 h in 48-well plates. CHIKV infection followed at an MOI of 0.01, and after 24 h, virus copy numbers were quantified from supernatant RNA. Western blot analysis was used to detect CHIKV E1 protein in lysed 293T cells. Additionally, BHK-21 cells were reinfected with 293T supernatants, and viral plaques were visualized with crystal violet after 48 h.

A time-of-addition assay determined the viral life cycle step targeted by SA. 293T cells were infected with CHIKV at an MOI of 0.01 for 2 h. Different concentrations of SA were introduced to the CHIKV-infected cells at distinct time intervals: pre-infection (−2 to 0 h), during infection (0–2 h), and post-infection (2–4 h). Following incubation, supernatants were collected at 16 h, and RNA extractions were performed to determine the virus copy number by RT-qPCR. The primer sequences were in Supplementary Table 1.

#### 2.3.4 Determination of proliferation-related targets of CHIKV

To determine whether SA resistance to CHIKV involves the IL-17 signaling pathway, we infected 293T cells with 0.1 MOI for 2 h, followed by incubation with 1,000 μM SA. RNA was extracted at 24 h post-infection to measure the expression levels of related targets in the IL-17 signaling pathway, including NFKBIA, PTGS2, MAPK3, RELA, EGF, and TLR2. Furthermore, molecular docking was conducted to validate the interaction between SA and IL-17 signaling pathway components, evaluating the binding affinity of SA to IL-17-related proteins: IL-17A, IL-17E, IL-17RA, IL-17RB, TRAF6, the Act1 binding alpha-helix, and the IL-25-IL-17RB-IL-17RA ternary complex.

To investigate the inhibitory effect of SA on the adsorption of CHIKV to host cell, we assessed the cytokines associated with CHIKV cell adsorption. In brief, 293T cells were treated with 1,000 μM SA for 2 h, followed by incubation with CHIKV at a MOI of 0.1. RNA was extracted at 24 h post-infection to measure the expression levels of ACTG1, FHL1, TIM-1, COL1A2, PTPN2, IFITM3, and TSPAN9.

Relative RNA levels were quantified using the 2^−ΔΔ*Ct*^ method. The primer sequences are listed in Supplementary Table 1.

#### 2.3.5 Statistical analysis

The experimental data were statistically analyzed using GraphPad Prism 9.50. Significance levels were determined using the unpaired Student's *t*-test for two-group comparisons or one-way analysis of variance (ANOVA) for multiple group comparisons, with statistical significance denoted by *p* < 0.05.

## 3 Results

### 3.1 Network pharmacology-based analysis

From multiple disease databases, a total of 706 unique disease-related targets were identified after consolidating data from DisGeNET (138 targets), CTD (292 targets), GeneCards (210 targets), MalaCards (33 targets), OMIM (86 targets), and STRING (111 targets); no targets were found in TTD. For compound searches related to SA, 423 targets were identified across CTD (9 targets), PharmMapper Server (285 targets), STRING (33 targets), and Swiss Target Prediction (100 targets), with no targets from CTD and TTD.

**Figure 1:**
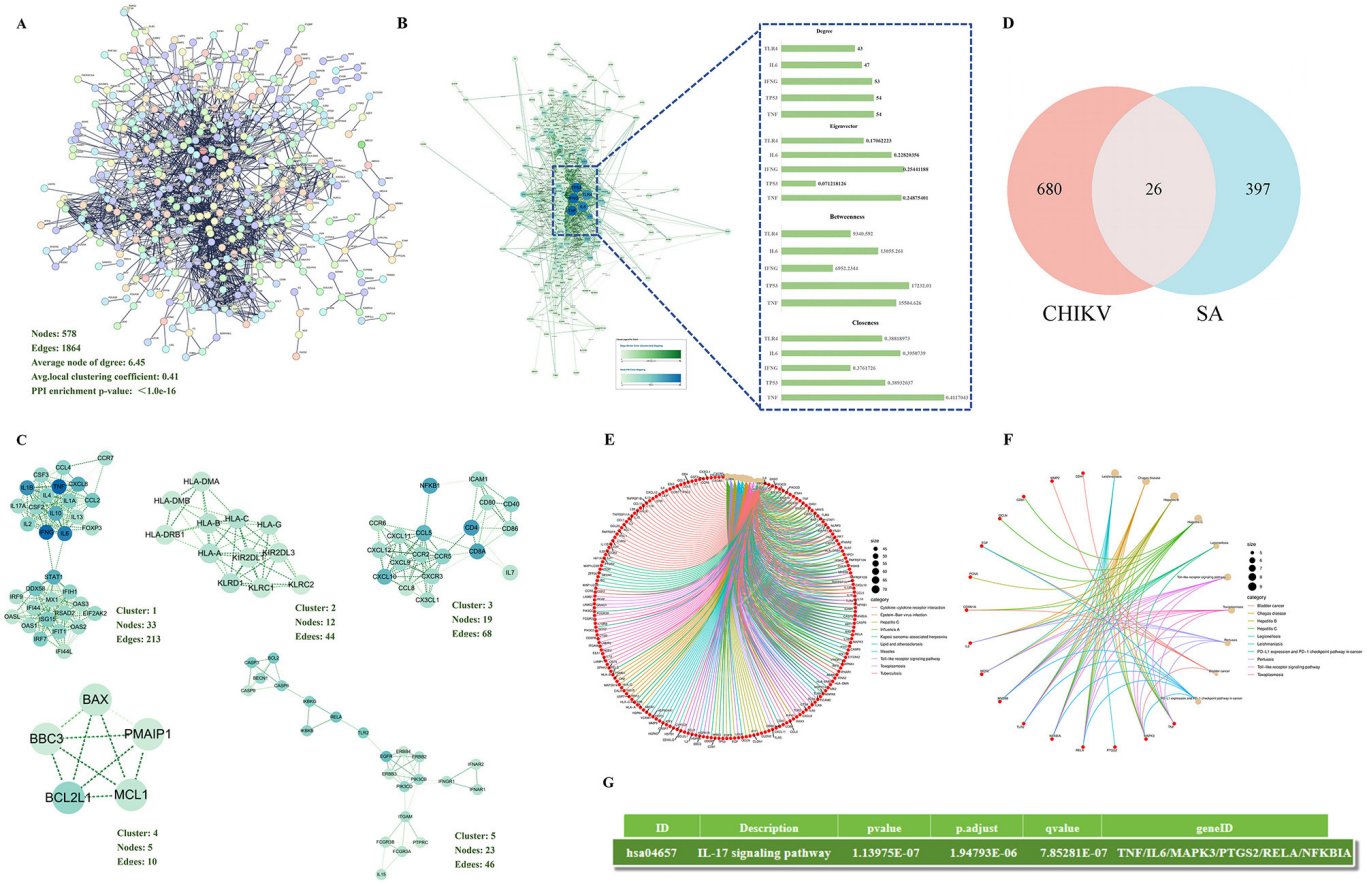
Network pharmacological analysis **(A)** The PPI network was constructed to visualize the connectivity among disease-related targets. **(B)** Core targets within the disease context were identified using four distinct algorithms within Cytoscape software. **(C)** The top five functional clusters among the disease-related targets were determined using the MCODE plugin in Cytoscape. **(D)** An intersection analysis between SA targets and disease-related targets revealed 26 common targets. **(E)** Bubble chart of KEGG pathway analysis of disease-related targets. **(F)** Bubble chart of KEGG pathway analysis of 26 common targets. **(G)** The target of SA against CHIKV is enriched in the interleukin-17 signaling pathway

Based on STRING analysis, a PPI network comprising 578 nodes and 1,864 edge was generated, which was significantly enriched (Figure 1A). Cytoscape 3.9.1 and the CytoNCA plugin were utilized to analyze the core targets of disease using four algorithms: Betweenness, Closeness, Degree, and Eigenvector. The top five core targets identified were TNF, TP53, IFNG, IL6, TLR4, and IL1B (Figure 1B). MCODE analysis indicated that cluster 1 was the primary pathogenic subcluster associated with CHIKV (Figure 1C). To identify common targets between diseases and SA, Venn analysis was employed, revealing 26 shared targets (Figure 1D).

A comprehensive analysis of the 706 disease-associated targets generated a dataset for KEGG pathway enrichment. This analysis identified 160 KEGG terms linked to the disease-related targets, with the top 10 pathways selected based on their lowest *p*-values (Figure 1E). The KEGG analysis results for common targets yielded 92 terms, with the top 10 pathways (Figure 1F). Furthermore, the results of the KEGG analysis, we also found that SA can regulate the IL-17 signaling pathway against CHIKV (Figure 1G).

### 3.2 Molecular docking

**Figure 2:**
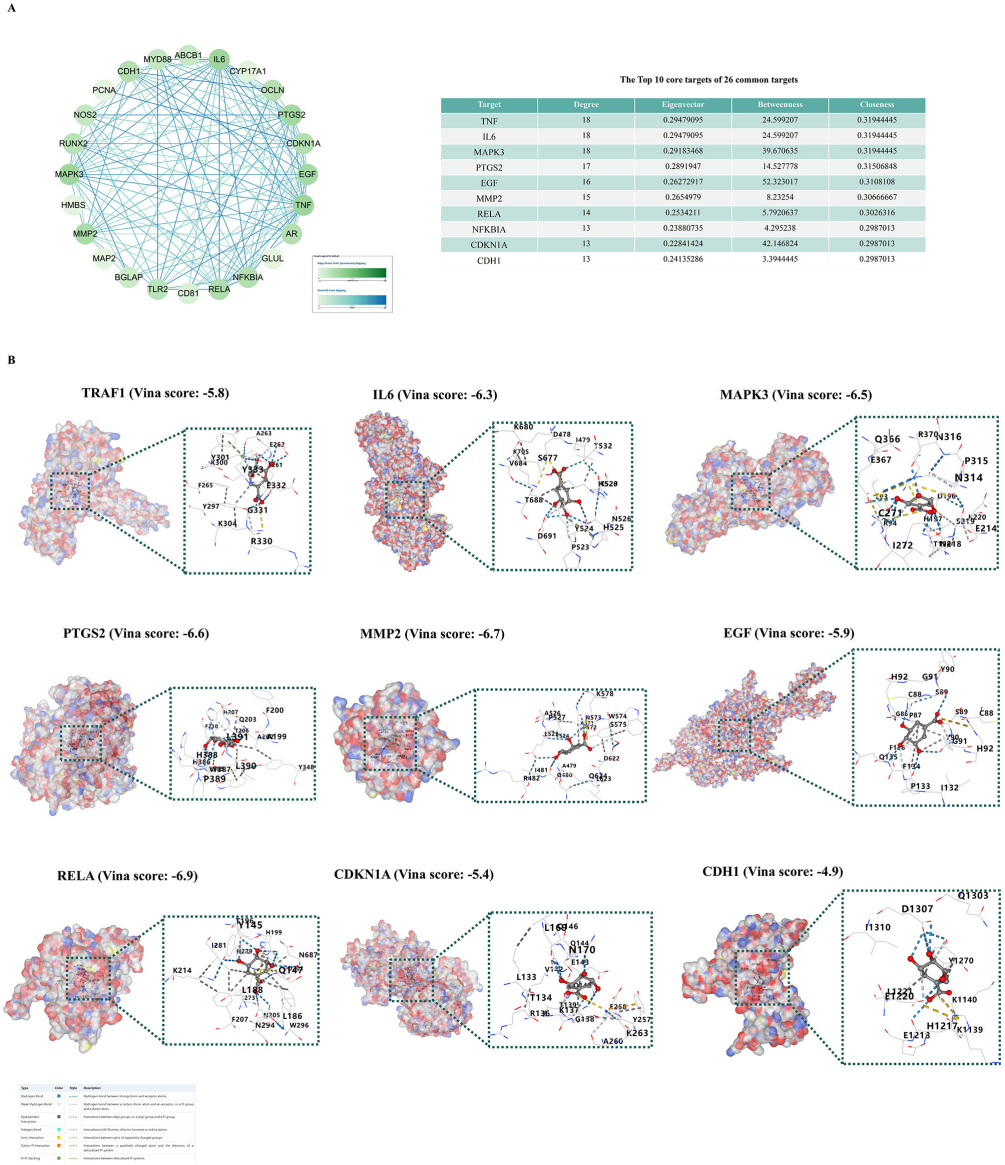
Molecular docking verification. **(A)** Core targets analysis of 26 common genes using cytoscape. **(B)** Molecular docking models of SA with nine protein targets

Based on the scores of the four algorithms of Cytoscape 3.9.1, we identified the top 10 targets with the highest scores among the 26 common targets for molecular docking (Figure 2A). We found nine of these targets in the PDB database according imitation of a crystal resolution below 3 and determined through X-ray crystal diffraction. The identified are TRAF1 (PDB ID: 5e1t), IL6 (PDB ID: 5sfk), MAPK3 (PDB ID: 4qtb), PTGS2 (PDB ID: 5f19), EGF (PDB ID: 5mwf), MMP2 (PDB ID: 5th6), RELA (PDB ID: 1rtg), CDKN1A (PDB ID: 7kys), CDH1 (PDB ID: 4b4c). Subsequently, we performed molecular docking of SA with each of these proteins individually and obtained Vina scores for SA's interaction with different proteins. The Vina score is a scoring function based on the force field, primarily calculating van der Waals forces and Coulombic interactions. In molecular docking results, a lower score indicates a higher affinity between the ligand and the receptor, suggesting a tighter binding of the ligand to the receptor. The results demonstrated that SA could stably bind to nine target proteins, with Vina scores consistently below −6 (Figure 2B).

### 3.3 Assessing the protective effect of SA on cell viability in CHIKV infection

**Figure 3:**
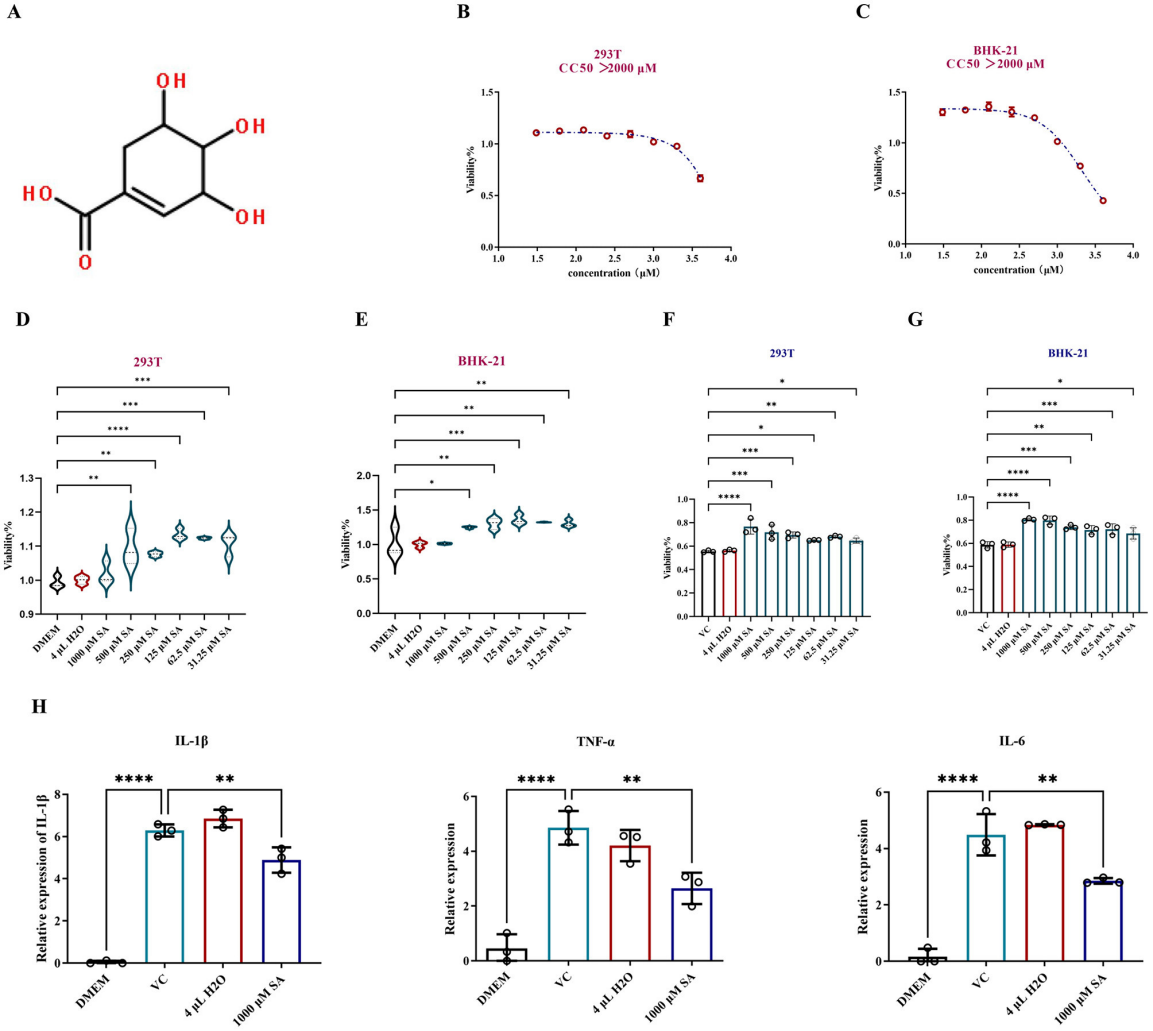
*In vitro* assessment of protection of SA against CHIKV-infected cells. **(A)** Chemical structure of SA. **(B)** Cytotoxic concentration 50% (CC50) of SA in 293T cells. **(C)** Cytotoxic concentration 50% (CC50) of SA in BHK-21 cells. **(D)** Impact of SA on 293T cell proliferation assessed by CCK-8 assay. **(E)** Impact of SA on BHK-21 cell proliferation assessed by CCK-8 assay. **(F)** Viability of SA in promoting 293T cells infected with CHIKV. **(G)** Viability of SA in promotingBHK-21 cells infected with CHIKV. **(H)** Expression levels of inflammatory cytokines IL-1β, TNF-α, and IL-6. DMEM: uninfected cell control group, VC: virus infection control group, 4 μl H_2_O: mock treatment group. *Compared with VC or DMEM. All values represent the mean ± SD. ****p < 0.0001, ***p < 0.001, **p < 0.01, *p < 0.05.

The chemical structure of SA is illustrated in Figure 3A. Cytotoxicity was evaluated using the CCK-8 assay with concentrations ranging from 4,000 to 31.25 μM. The CC50 of SA exceeded 2,000 μM for both cell types, establishing a non-toxic threshold of 1,000 μM established as the maximum safe concentration (Figures 3B, C). A proliferation-enhancing effect of SA was observed in 293T and BHK-21 cells (Figures 3D, E).

To assess the protective effect of SA on CHIKV-infected cells, dilutions starting at 1,000 μM were with a two-fold reduction were prepared and incubated with CHIKV in 293T and BHK-21 cells for 24 h. Figures 3F, G indicate that SA significantly improved cell viability in the presence of CHIKV at a 0.01 MOI, exhibiting a dose-dependent protective effect. RT-qPCR analysis of RNA from CHIKV infected-293T cells treated with SA revealed a significant reduction in the expression levels of inflammatory cytokines IL-1β, TNF-α, and IL-6 (Figure 3H).

### 3.4 *In vitro* antiviral effect evaluation

**Figure 4:**
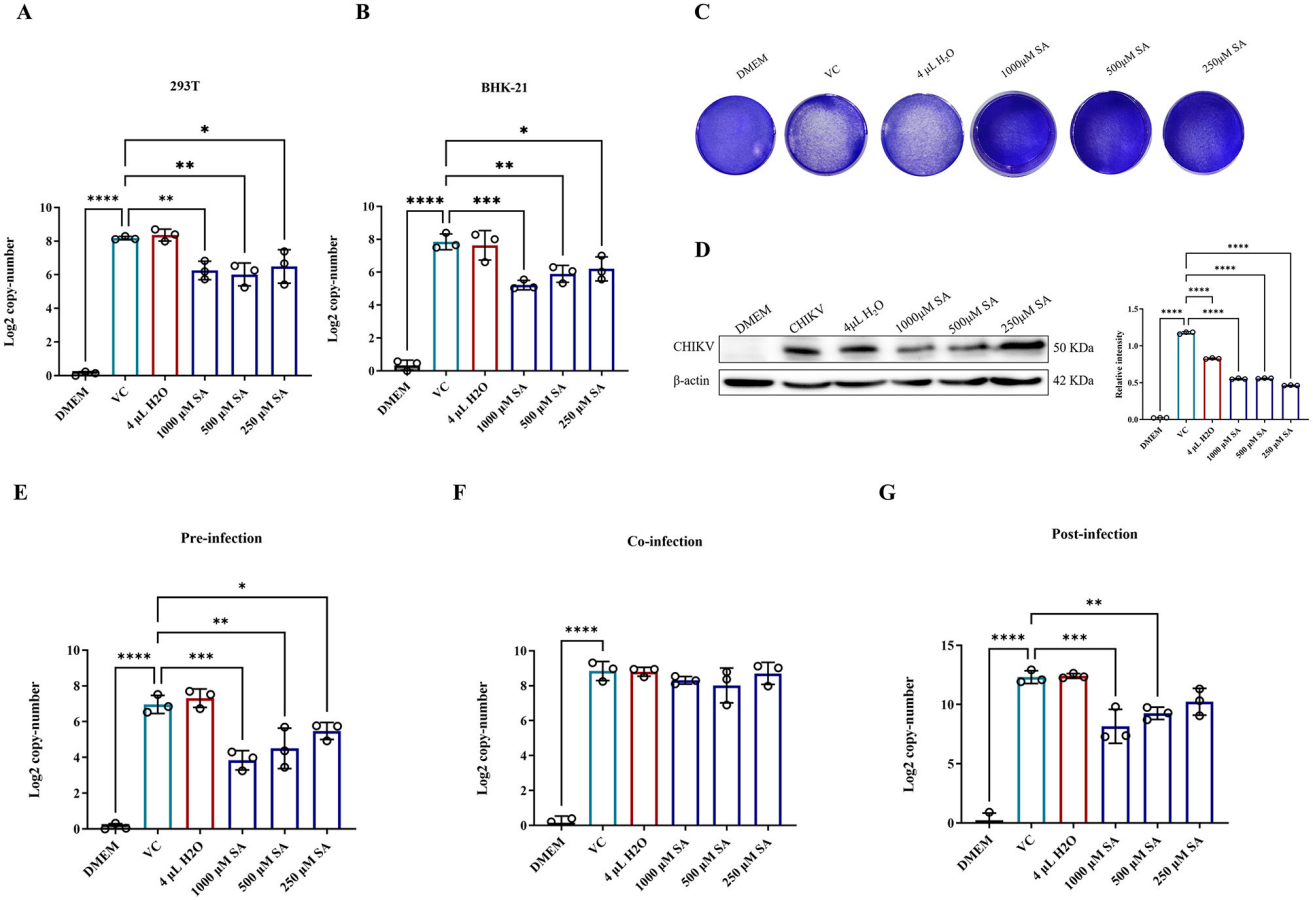
*In vitro* antiviral activity of SA against CHIKV. **(A)** CHIKV copy number in 293T cells after treatment, as determined by RT-PCR. **(B)** CHIKV copy number in BHK-21 cells after treatment, as determined by RT-PCR. **(C)** Crystal violet staining of cells to assess cytopathic effects **(D)** Western blot analysis of CHIKV E1 protein expression in 293T cells. **(E)** CHIKV copy number in 293T cells pre-infection. **(F)** CHIKV copy number in 293T cells during co-infection. **(G)** CHIKV copy number in 293T cells post-infection. DMEM: uninfected cell control group, VC: virus infection control group, 4 μl H_2_O: mock treatment group. Compared with VC. All values represent the mean ± SD. ****p < 0.0001, ***p < 0.001, **p < 0.01, *p < 0.05.

The inhibitory effect of SA on CHIKV replication was evaluated by pretreating 293T and BHK-21 cells with SA concentrations from 1,000 to 250 μM for 2 h before CHIKV infection at a MOI of 0.01. The results indicated a significant reduction in virus copy number by ~25% in both cell lines (Figures 4A, B). Crystal violet staining revealed a decrease in the number of CHIKV plaques in BHK-21 cells at these concentrations (Figure 4C). Western blot analysis also significantly reduced CHIKV E1 protein levels in 293T cells (Figure 4D).

A time-of-addition assay was conducted to explore the life cycle of SA-induced CHIKV inhibition. 293T cells were treated with SA at concentrations from 1,000 to 250 μM at different stages of the viral life cycle, with copy numbers measured at 16 h post-infection. The results showed that SA significantly reduced the CHIKV copy number during pre-infection and post-infection periods (Figures 4E–G). However, during co-infection, these concentrations did not significantly inhibit the CHIKV copy number (Figure 4F).

### 3.5 Determination of proliferation-related targets of CHIKV

**Figure 5:**
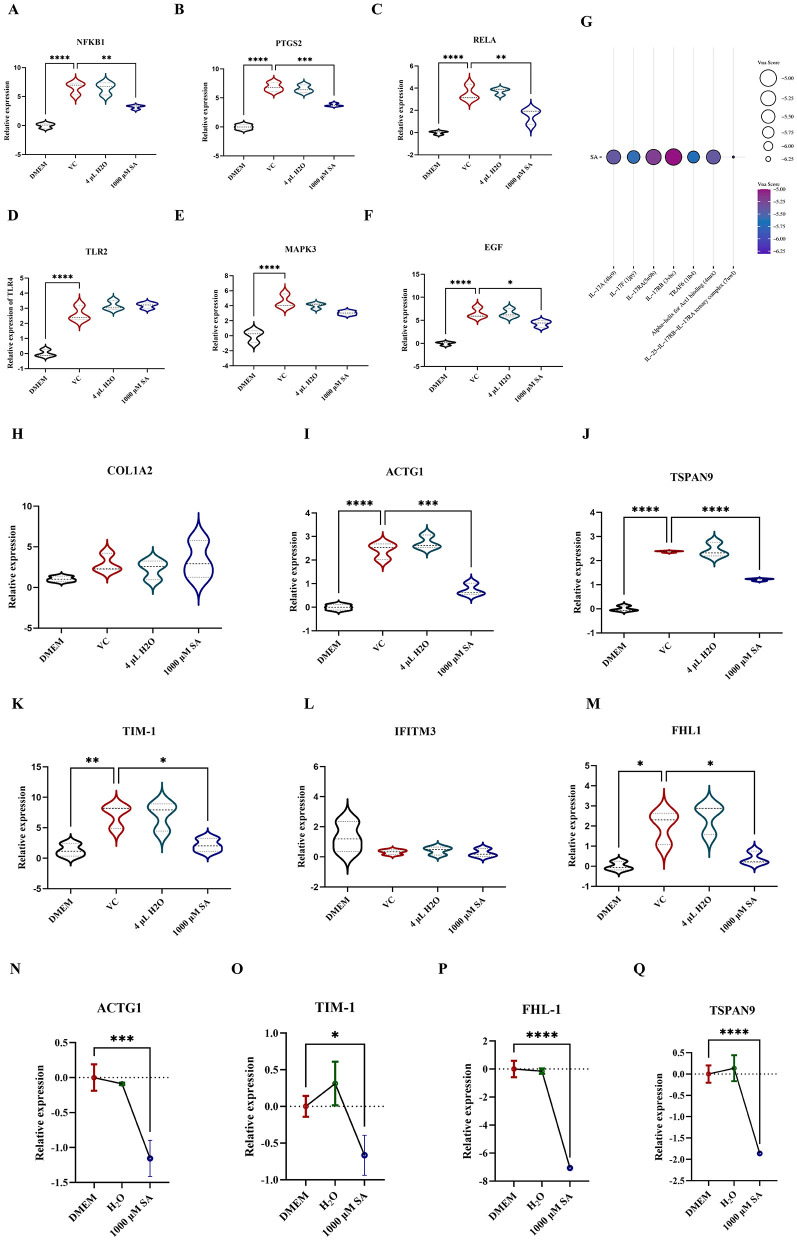
Detection of relevant targets of SA against CHIKV replication **(A)** The relative expression level of NFKB1 in 293T cells with infected with and without CHIKV. **(B)** The relative expression level of PTGS2 in 293T cells with infected with and without CHIKV. **(C)** The relative expression level of RELA in 293T cells with infected with and without CHIKV. **(D)** The relative expression level of TLR2 in 293T cells with infected with and without CHIKV. **(E)** The relative expression level of MAPK3 in 293T cells with infected with and without CHIKV. **(F)** The relative expression level of EGF in 293T cells with infected with and without CHIKV. **(G)** Molecular docking of SA with proteins associated with IL-17 signaling pathway. **(H)** The relative expression level of COL1A2. **(I)** The relative expression level of ACTG1 **(J)** The relative expression level of TSPAN9. **(K)** The relative expression level of TIM-1. **(L)** The relative expression level of IFITM3. **(M)** The relative expression level of FHL-1. **(N)** The relative expression level of ACTG1 in 293T cells in 293T cells treated with and without SA. **(O)** The relative expression level of TIM-1 in 293T cells in 293T cells treated with and without SA. **(P)** The relative expression level of FHL-1 in 293T cells in 293T cells treated with and without SA. **(Q)** The relative expression level of TSPAN9 in 293T cells in 293T cells treated with and without SA. DMEM: uninfected cell control group, VC: virus infection control group, 4 μl H_2_O: mock treatment group. All values represent the mean ± SD. Compared with VC or DMEM. All values represent the mean ± SD. ****p < 0.0001, ***p < 0.001, **p < 0.01, *p < 0.05.

To explore the association between the antiviral activity of SA against CHIKV and the IL-17 signaling pathway, the expression levels of key genes were quantified via RT-qPCR. It was found that 1,000 μM SA significantly downregulated the expression of NFKB1 (Figure 5A), PTGS2 (Figure 5B), RELA (Figure 5C), and EGF (Figure 5F). However, no significant changes were detected for TLR2 and MAPK3 (Figures 5D, E). Molecular docking studies confirmed interactions between SA and the IL-17 signaling pathway, with SA exhibiting the highest binding affinity to the IL-25-IL-17RB-IL-17RA complex and TRAF6, as indicated by Vina scores of −6.3 and 5.7, respectively (Figure 5G).

The influence of SA on CHIKV attachment factor expression was assessed by extracting RNA from 293T cells and conducting RT-qPCR to measure ACTG1, FHL1, TIM-1, COL1A2, PTPN2, IFITM3, and TSPAN9 levels (Figures 5H–M). SA was found to reduce the expression of ACTG1 (Figure 5I), TSPAN9 (Figure 5J), TIM-1 (Figure 5K), and FHL-1 (Figure 5M) in CHIKV-infected 293T cells, with no significant alterations in COL1A2 and IFITM3 expression. Further validation via RT-qPCR on RNA from uninfected 293T cells confirmed that 1,000 μM SA suppressed the expression of ACTG1, TIM-1, FHL-1, and TSPAN9 (Figures 5N–Q).

## 4 Discussion

Utilizing network pharmacology to delineate the interactions between compounds and diseases significantly reduces the costs and associated with trial-and-error approaches. Since the efficacy of SA against CHIKV had not been previously established, this study employed a network pharmacology approach to evaluate the potential of SA against CHIKV, a hypothesis further supported by molecular docking analyses. Our results indicate that SA can modulate the expression of inflammatory factors following CHIKV infection, a finding that was validated through subsequent cell-based assays. The inflammatory biomarkers TNF-α and IL-6 are associated with increased severity of CHIKV infection and pain (13, 14). The prolonged presence of cytokines leads to persistent multi-joint pain, impeding daily activities (15, 16). *In vitro* infection models demonstrated that SA reduced TNF-α and IL-6 expression in CHIKV-infected 293T cells and increased the survival rate of infected BHK-21 and 293T cells by ~25%. These findings not only validate the potential of SA as an antiviral agent against CHIKV but also hint at its potential to alleviate pain by downregulating the expression of inflammatory factors. However, this latter possibility, while speculative based on previous research outcomes, requires *in vivo* verification.

*In vitro* experiments revealed an interesting phenomenon, a concentration of 1,000 μM SA was found to be optimal for protecting cells from CHIKV infection without promoting the growth of uninfected cells. This observation suggests that the protective effect of SA is mediated through an antiviral mechanism rather than through the promotion of cell proliferation. To verify this hypothesis, we quantified viral genome copy numbers in the culture medium of CHIKV-infected 293T cells treated with SA at concentrations ranging from 1,000 to 250 μM. Our findings demonstrated that SA significantly reduced the production of new viral particles within this concentration range Furthermore, western blot analysis revealed a marked reduction in E1 protein levels in CHIKV-infected 293T cells treated with SA. A time-of-addition assay showed that SA treatment, whether administered before or after virus exposure, significantly reduced CHIKV proliferation within cells; however simultaneous addition with the virus did not inhibit replication. This suggests that SA inhibits CHIKV during the stages of viral adsorption and intracellular replication. For CHIKV to initiate infection, it must adhere to host cells. The CHIKV surface is adorned with ~80 spike-like envelope (E) proteins, which are organized as trimers of E1/E2 heterodimers (17). The E1 protein is crucial for the fusion of viral and cellular membranes, while the E2 protein engages with host receptors to facilitate viral entry (18). Host factors, including TIM-1, FHL1, and TSPAN9, are known to facilitate CHIKV entry and promote infection (19–21), whereas others, such as IFITM3, inhibit viral adsorption by limiting membrane fusion and virion internalization (22). Certain host factors, including ACTG1, PTPN2, and COL1A2, may interact with E2 proteins, potentially facilitating or impeding cell entry (23). To assess the impact of SA on the expression of these host factors, we employed RT-qPCR. The results demonstrated that SA reduced the expression of ACTG1, TIM-1, FHL1, and TSPAN9 in both CHIKV-infected and non-infected 293T cells. These findings suggest that SA has the potential to modulate the expression of these factors, which may impede CHIKV adsorption to 293T cells.

Using network pharmacology, we identified six targets involved in the anti-CHIKV activity of SA that were significantly enriched in the IL-17 signaling pathway. The IL-17 family plays a crucial role in defending against microbial invasions and in the pathogenesis of inflammatory diseases (24). The IL-17 signaling pathway is activated when IL-17A or IL-17E (also known as IL-25) binds to the IL-17 receptor, leading to the recruitment of Act1 to IL-17RA and/or IL-17RC via its SEFIR domain (25–27). This interaction triggers the recruitment of TRAF6 and activates key transcription factors such as NF-κB, MAPK-AP-1, and C/EBP, which are central to CHIKV pathogenesis. Activation of this pathway induces pro-inflammatory genes, including IL-6, and stimulates the RANKL system (28). Elevated IL-6 levels can activate RANKL and suppress the release of osteoprotegerin, resulting in increased bone damage (29). IL-17A-deficient mice infected with CHIKV exhibited reduced tissue inflammation and neutrophil infiltration compared to wild-type mice (30), suggesting that targeting the IL-17 pathway could alleviate CHIKV symptoms. Furthermore, the IL-17 signaling pathway is associated with CHIKV proliferation, as IL-17A has been shown to inhibit CHIKV-induced IFN-α2 expression and enhance CHIKV replication in cells (31). KEGG enrichment analysis in this study revealed that SA modulates IL-17 activation in response to CHIKV. We assessed the expression of IL-17-related cytokines identified via network pharmacology using RT-qPCR. The results indicated that SA significantly reduced the expression of IL-17 signaling pathway-associated factors, including NFKB1, PTGS2, RELA, TLR4, MAPK3, and EGF, in 293T cells infected with CHIKV. Moreover, molecular docking studies suggested that SA might inhibit the IL-17 signaling pathway by blocking the interaction between the IL-25-IL-17RB-IL-17RA complex and TRAF6. By inhibiting this interaction, SA could potentially disrupt the signaling cascade leads to inflammation and tissue damage during CHIKV infection and inhibit CHIKV replication in cells.

In conclusion, our study suggests that SA may be a promising compound for treating CHIKV infection. However, acknowledging the limitations of our research is crucial; it was limited to *in vitro* assessments of SA's impact on CHIKV. The *in vivo* antiviral efficacy and the underlying mechanisms of SA against CHIKV remain to be determined.

## Data Availability

The datasets presented in this study can be found in online repositories. The names of the repository/repositories and accession number(s) can be found in the article/Supplementary material.
