# Supplementary material for: Exploring the antiviral potential of shikimic acid against Chikungunya virus through network pharmacology, molecular docking, and in vitro experiments
Source: Front Vet Sci. 2025 Jan 23;12:1524812. doi: 10.3389/fvets.2025.1524812 (PMC11799295; doi:10.3389/fvets.2025.1524812)
Supplement: Supplementary file 1 [file Table_1.docx]

Supplementary Table 1. Primer pairs used for target validation

| Primers | Sequences |
| --- | --- |
| Nsp2 | F：5′-GGCAGTGGTCCCAGATAATTCAAG-3′ |
|  | R：5′-GTACATACCCCACCTAGATCTGTCG-3′ |
| β-Actin | 5′-ATCACCATTGGCAATGAGCG-3′ |
|  | 5′-TTGAAGGTAGTTTCGTGGAT-3′ |
| NFKBIA | F：5′-CTCCGAGACTTTCGAGGAAATAC-3′ |
|  | R：5′-GCCATTGTAGTTGGTAGCCTTCA-3′ |
| PTGS2 | F：5′-CTGGCGCTCAGCCATACAG-3′ |
|  | R：5′-CGCACTTATACTGGTCAAATCCC-3′ |
| MAPK3 | F：5′-CTACACGCAGTTGCAGTACAT-3′ |
|  | R：5′-CAGCAGGATCTGGATCTCCC-3′ |
| RELA | F：5′-ATGGGCAAGTCAGCTTCCAAA-3′ |
|  | R：5′-GCCTCAGAATACTGTTGAGCCT-3′ |
| EGF | F：5′-AGACCTACGACGGGAGCAC-3′ |
|  | R：5′-ACCTGCATTAGAGCAGATGATGT-3′ |
| TLR2 | F：5′-ATCCTCCAATCAGGCTTCTCT-3′ |
|  | R：5′-GGACAGGTCAAGGCTTTTTACA-3′ |
| ACTG1 | F：5′-CCGAGCCGTGTTTCCTTCC-3′ |
|  | R：5′-GCCATGCTCAATGGGGTACT-3′ |
| FHL1 | F：5′-TGCTGCCTGAAATGCTTTGAC-3′ |
|  | R：5′-GCCAGAAGCGGTTCTTATAGTG-3′ |
| TIM-1 | F：5′-AACTGTCTCTACCTTGTTCCTCC-3′ |
|  | R：5′-GTTCTCTCCTTATTGCTCCCTG-3′ |
| COL1A2 | F：5′-GTGGCAGTGATGGAAGTGTG-3′ |
|  | R：5′-AGGACCAGCGTTACCAACG-3′ |
| PTPN2 | F：5′-GCAGTGAGAGCATTCTACGGA-3′ |
|  | R：5′-TGACACAAACCCCATCTTAGTGA-3′ |
| IFITM3 | F：5′-GACCATTCTGCTCATCGTCATC-3′ |
|  | R：5′-AGCCAGACCCTCCCAATGTT-3′ |
| TSPAN9 | F：5′-AACGAGAACGCCAAGAAGGA-3′ |
|  | R：5′-CGTTGTTCTCGGTGTGGTACA-3 |
